# Dr. Robert Harrison

**Published:** 1858-07

**Authors:** 


ON THE same day, the Medical Profession of Ireland sustained a great loss in the death, by apoplexy, of DR. ROBERT HARRISON, Professor of Anatomy in Trinity College, Dublin. For forty years, and up to the day of his death, he was an able, fluent, and popular teacher of his branch, and for a long while well known by his treatise on the "Surgical Anatomy of the Arteries," which, at the time of its publication, about 1830, was the best production of the kind in the English language. He was also the author of the well-known "Dublin Dissector."

